# Multi-omics association study integrating GWAS and pQTL data revealed MIP-1α as a potential drug target for erectile dysfunction

**DOI:** 10.3389/fphar.2024.1495970

**Published:** 2024-11-01

**Authors:** Jingwen Liu, Renbing Pan

**Affiliations:** ^1^ Longyou People’s Hospital Affiliated with Sir Run Run Shaw Hospital, Zhejiang University School of Medicine, Quzhou, Zhejiang, China; ^2^ Department of Urology, The Quzhou Affiliated Hospital of Wenzhou Medical University, Quzhou People's Hospital, Quzhou, Zhejiang, China

**Keywords:** erectile dysfunction, drug target, GWAS, multi-omics, MIP-1α

## Abstract

**Background:**

Erectile dysfunction (ED) brings heavy burden to patients and society. Despite the availability of established therapies, existing medications have restricted efficacy. Therefore, we utilized a two-sample Mendelian randomization (MR) approach to find the drug targets that might enhance the clinical outcome of ED.

**Methods:**

Genetic instruments associated with circulating inflammatory proteins were obtained from a genome-wide association study (GWAS) involving 8,293 European participants. Summary statistics for ED were extracted from a meta-analysis of the United Kingdom Biobank cohort compromised of 6,175 cases and 217,630 controls with European descent. We utilized multi-omics method and MR study to explore potential drug targets by integrating GWAS and protein quantity trait loci (pQTL) data. Inverse-variance weighted (IVW) method was applied as the primary approach. Cochran’s Q statistics was employed to investigate the presence of heterogeneity. Furthermore, we identify the potential therapeutic drug targets for the treatment of ED utilizing molecular docking technology.

**Results:**

This MR analysis of integrating GWAS and pQTL data showed that macrophage inflammatory protein-1 alpha (MIP-1α) was causally associated with the risk of ED (OR:1.19, 95%CI:1.02–1.39, *p* = 0.023). Meanwhile, the results of the weighted median model were consistent with the IVW estimates (OR:1.26, 95%CI:1.04–1.52, *p* = 0.018). Sensitivity analysis revealed no horizontal pleiotropy and heterogeneity. Furthermore, four anti-inflammatory or tonifying small molecular compounds, encompassing echinacea, pinoresinol diglucoside, hypericin, and icariin were identified through molecular docking technology.

**Conclusion:**

This study identified MIP-1α as an underlying druggable gene and promising novel therapeutic target for ED, necessitating further investigation to detect the potential mechanisms by which MIP-1α might impact the development of ED.

## 1 Introduction

To our knowledge, the term erectile dysfunction (ED) refers to the inability to achieve and sustain a sufficient penile erection for satisfactory sexual intercourse (Bacon et al., 2003; Bohm et al., 2007). The condition is prevalent among males in the middle-aged and elderly populations (Shiri et al., 2006), with an estimated prevalence rate ranging from 25% to 35% (Gleason et al., 2011). The projected global incidence of ED is expected to reach 322 million cases by 2025, as per forecasts (Ayta et al., 1999). Therefore, the prevention and early intervention for the risk factors of ED are of significant importance and require a sense of urgency.

The pathophysiology of ED involves the interplay of various factors, encompassing vasculogenic, neurogenic, hormonal, iatrogenic, anatomical, and psychogenic influences (Corona et al., 2006). Several studies show that the presence and seriousness of ED are related to endothelial dysfunction and markers and mediators of subclinical inflammation (Vlachopoulos et al., 2007). Previous studies show that patients with ED exhibit elevated levels of inflammatory markers, encompassing adhesion molecules, inflammatory cytokines, and endothelial-prothrombotic markers (Vlachopoulos et al., 2006; Chiurlia et al., 2005; Arana Rosainz Mde et al., 2011; Bocchio et al., 2004; Meldrum et al., 2012; Esposito et al., 2005). The severity of ED has been consistently associated with the regulation of inflammation, as demonstrated by numerous clinical and preclinical studies. Circulating inflammatory proteins play a significant role in the pathogenesis of ED, exerting both beneficial and deleterious effects. Suvanlli et al. reported that plasma levels of high-sensitivity CRP and fibrinogen exhibited an elevation in patients with ED when compared to individuals without ED (Sullivan et al., 2001). Another study by Zuo et al. revealed that tumor necrosis factor (TNF-α) inhibits the expression of endothelial NO synthase in endothelial cells (Anderson et al., 2004; Neumann et al., 2004; Yoshizumi et al., 1993). The injection of senescent cells, which produced proinflammatory-cytokines TNF-α and interleukin (IL)-1β, resulted in impaired erectile response in mouse models by inducing endothelial dysfunction and nerve injury (Nishimatsu et al., 2015). Previous evidence suggests that there is an inverse correlation between the International Index of Erectile Function 5(IIEF-5) score, which measures sexual performance, and circulating inflammatory parameters, such as IL-6 and IL-1 (Vlachopoulos et al., 2006). The serum levels of inflammatory factors such as IL-1, IL-2, and IL-10 in type 2 diabetic mellitus rats with ED were significantly elevated compared to those in control rats during preclinical animal experiments (Li et al., 2017). These aforementioned studies emphasize the pathophysiological involvement of inflammatory proteins and endothelial dysfunction in the etiology of ED. Nevertheless, whether inflammation factors are the cause of ED or due to drug usage after disease progression remains controversial. Further studies are essential to elucidate the causal associations between systemic inflammatory proteins and ED.

Concerning the susceptibility of observational studies to lack control bias due to underlying confounders and reverse causality (Zhao G. et al., 2023), Mendelian randomization (MR) was employed to evaluate the causality between exposure and outcome. This analysis approach, based on genome-wide association studies (GWAS), is a robust method for constructing instrumental variables (IVs) and inferring causal effects between exposure and outcome in situations where establishing causality is challenging due to the retrospective and observational nature of the study (Ren et al., 2023). The evidence of causality provided by the MR research was reported to lie at the interface between conventional epidemiological studies and randomized controlled trials (RCTs) (Chen et al., 2023). For instance, a bidirectional MR study found that targeted interventions of specific inflammatory proteins could mitigate the risk of meningiomas (Zhang et al., 2023). In addition, another MR study suggested that genetically increased specific inflammatory regulator levels demonstrated a protective effect against the risk of ischemic stroke (Li et al., 2021). In view of the correlations between inflammatory proteins and ED and the clinical efficacy of traditional Chinese medicine (TCM) on ED, we speculate whether certain TCM monomeric compounds can achieve efficacy in the treatment of ED by interfering with these specific inflammatory proteins. Therefore, in this study, we utilized multi-omics approach and MR study to explore potential drug targets by integrating GWAS and protein quantity trait loci (pQTL) data. Additionally, through the utilization of molecular docking technology, we screen the eligible TCM monomeric compounds for regulating inflammation in the treatment and prevention of ED.

## 2 Materials and methods

### 2.1 Study design and assumptions

**FIGURE 1:**
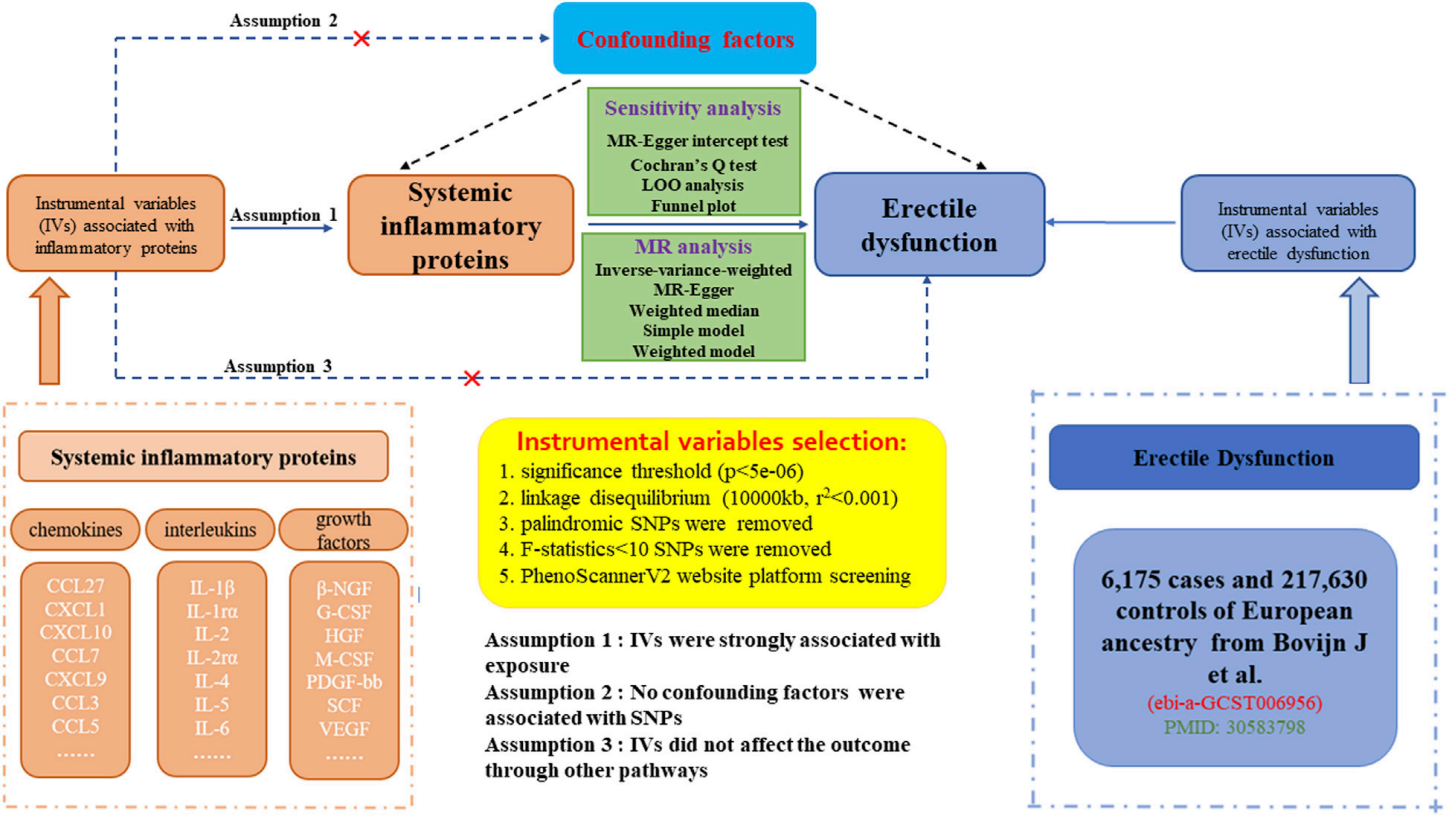
Datasets, flowchart, and study design of two-sample Mendelian randomization for systemic inflammatory proteins and erectile dysfunction (ED).

The workflow of our study was displayed in Figure 1. The study was on the basis of publicly available datasets from GWASs on systemic inflammatory proteins and ED, with explicit information provided in Supplementary Table S1. The explicit process encompassed three steps: (1) eligible single nucleotide polymorphisms (SNPs) associated with 41 circulating inflammatory proteins were extracted according to the specified threshold standard. (2) the two-sample MR method was employed to detect the links between systemic inflammatory proteins and ED one by one. (3) sensitivity analysis was performed on the results of MR estimates. To augment the reliability of the MR analysis findings, our study endeavored to fulfill the following three assumptions. (1) the IVs are strongly associated with circulating levels of inflammatory proteins. (2) no confounding factors are associated with the IVs. (3) the IVs affect the outcome only through exposure and there are no other causal pathways for the IVs to influence the outcome (Liang et al., 2023). We extracted genetic IVs for each circulating inflammatory protein to explore the causal link from each inflammatory protein to ED. Lastly, we use molecular docking techniques to screen potential therapeutic targets for significant inflammatory proteins. The utilization of publicly available GWAS summary datasets obviated the need for ethical approval.

### 2.2 Data sources

Exposure: We collected genome-wide association summary statistics for 41 circulating inflammatory proteins (pQTL) from the most proximate GWAS reported by Ahola-Olli et al. (2017), encompassing up to 8,293 Finnish individuals from three independent cohorts: FINRISK 1997, FINRISK 2002, and the Cardiovascular Risk in Young Finns Study (YFS), These inflammatory protein distributions were normalized by converse transformation. An additive genetic model, adjusted for sex, age, body mass index (BMI), and the first ten genetic principal components, was employed to examine univariable correlations between 10.7 million genetic polymorphisms and the circulating levels of 41 inflammatory proteins.

Outcome: The summary data of ED was obtained from a large-scale GWAS meta-analysis comprising three cohorts exclusively consisting of European participants (6,175 cases and 217,630 controls) (Bovijn et al., 2019). This comprehensive study recruited 223,805 European males from the Partners Healthcare Biobank, the Estonian Genome Center of the University of Tartu, and the United Kingdom Biobank. ED cases were identified by doctor-diagnosed, self-report, or taking ED drugs. The absence of overlap occurred due to the acquisition of samples from diverse consortiums for both inflammatory proteins and ED.

### 2.3 Selection of genetic IVs

Firstly, the genome-wide significant threshold of *P* < 5e-08 was applied to each of the 41 inflammatory proteins to identify robust IVs associated with their levels. Due to no or few (<3) IVs being extracted for a majority of the inflammatory proteins at the *P*-value < 5e-08 level, we widen the threshold to *P*-value < 5e-06 to select eligible IVs. Eventually, all 41 inflammatory proteins were identified under this standard. Secondly, the impact of robust linkage disequilibrium (LD) between single nucleotide polymorphisms (SNPs) was mitigated by applying a LD threshold for the selected SNPs(*r* ^2^ < 0.001,10,000 kb), ensuring independence among instrumental variables for each exposure. Thirdly, to avoid weak instrument bias, the average of SNPs F-statistics was calculated, and the F-statistics>10 were regarded as strong IVs for our study (Jin et al., 2024). The F-statistic is a statistical measure that quantifies both magnitude and precision of the genetic impact on the trait. It can be calculated as F = *R* ^2^(N-2)/(1-R^2^), where *R* ^2^ represents the proportion of variance in the trait explained by the IVs, and N denotes the sample size of GWAS involving SNPs with the trait (Zhao B. et al., 2023). Lastly, intermediate allele frequency palindromic SNPs were excluded because allele frequencies were not provided in the GWAS of circulating inflammatory proteins, so we could not determine whether these SNPs were consistent with the direction of exposure and outcome.

### 2.4 Statistical analysis

To detect the causal estimations of inflammatory proteins on the risk of ED by conjoining various SNPs, we conducted a two-sample Mendelian randomization analysis employing five commonly analytical approaches, encompassing MR-Egger, weighted median, inverse variance weighted (IVW), simple mode, and weighted mode (Zheng et al., 2023). The random-effects IVW approach is the primary statistical method for aggregating Wald ratio estimations for diverse SNPs, exhibiting the highest statistic power among different MR methods. This approach was regarded as the primary analytical means of assessing the underlying causal associations between inflammatory proteins and ED. Our analysis necessitated data on SNPs, alleles, effect sizes, *P*-values, and allele frequencies (EAF) (Choi et al., 2019). The weighted median estimator could yield reliable estimation even when incorporating 50% of the invalid genetic instruments. Since the cutoff condition is widened, the MR-Egger approach yields a reliable estimation even in the presence of horizontal pleiotropy across SNPs, where all instrumental variables are invalid. Additionally, simple mode and weighted mode were also applied as auxiliary analytical methods.

### 2.5 Sensitivity analyses

The sensitivity analyses were performed by MR pleiotropy residual sum and outlier (MR-PRESSO), MR-Egger regression analysis, Cochran’s Q analysis, and leave-one-out (LOO) sensitivity analysis. Firstly, we utilized the simple-mode and weighted-median method to estimate the underlying causal effects in situations where conventional assumptions were challenged (Burgess et al., 2017). Secondly, The MR-Egger regression was conducted to assess the presence of horizontal pleiotropy, with statistical significance defined as *P*-values for the intercept being less than 0.05 (Chang et al., 2023). Thirdly, the MR-PRESSO global test was utilized to investigate potential outliers as plausible pleiotropic biases and mitigate the impact of pleiotropy by excluding the identified SNPs that deviated from normality. Finally, Cochran’s Q statistic was utilized for IVW and MR-Egger to assess heterogeneity among the estimates. A *P*-value greater than 0.05 in the Cochran’s Q test indicated the absence of heterogeneity among the IVs (Burgess et al., 2017). Additionally, we further examined whether some SNPs could affect the results independently and evaluated the stability of effect sizes via LOO sensitivity analysis. Furthermore, the Bonferroni correction was applied to address the issue of multiple comparisons, and a significance level of *P* < 0.0012 (0.05/41) was adopted (Bonferroni correction with 41 tests). All the MR analysis and sensitivity analysis were conducted with R (version 4.2.3). R packages “Two Sample MR” and “MR-PRESSO” packages were utilized.

### 2.6 Molecular docking technology

With RCSD Protein Data Bank (http://www.pdb.org), we acquired the crystal structures of targets (Deng et al., 2022). PyMOL software was utilized to isolate the primary ligand from the target protein and eliminate extraneous water molecules, phosphates, and other inactive ligands associated with the target protein (Warnecke et al., 2014). The 3D structural files of ligands were obtained from TCMSP, and with Chem3D software, their energy was minimized. The protein and active components were stored in the *pdb* format, while the AutoDockTools 1.5.6 software was employed to convert the *pdb* format of both the active components and proteins into the *pdbqt* format. The parameters for the active pocket were configured, and AutoDock was employed for docking. The active component was deemed to exhibit favorable target binding activity when the binding energy reached or fell below −5.0 kJ/mol (Tao et al., 2022). Discovery Studio 2019 was utilized to visualize the docking results.

### 2.7 Mapping candidate SNPs to genes and protein-protein interaction (PPI) network analysis

We employed the website platform PhenoScannerV2 (http://www.phenoscanner.medschl.cam.ac.uk/) for functional annotation of SNPs associated with eligible inflammatory proteins. Additionally, we visualized and predicted molecular interaction and PPI network utilizing STRING and GeneMANIA database. The degree algorithm of STRING software was employed to rank the significant proteins in PPI networks. GeneMANIA database can provide a protein-protein interactive network on gene and protein pathways, co-localization, co-expression, and functional assays with a pinpoint accuracy of prediction algorithm (Warde-Farley et al., 2010).

## 3 Results

### 3.1 Participant characteristics and genetic instruments

Considering the limited genetic variance as well as the restricted number of SNPs and low statistic powers, we conducted MR analysis by broadening the threshold to *P*-value < 5e-06. Finally, by using this cutoff condition (*r* ^2^ < 0.001, *P* < 5e-06) and removing the palindromic SNPs, altogether 455 SNPs (pQTL) associated with 41 inflammatory proteins were authenticated as IVs in our study. The F-statistics of IVs used in this study ranged from 20.77 to 345, suggesting that weak instrument bias may not be substantial. The figure and F-statistics of the SNPs which are utilized in this study are listed in Supplementary Table S2.

### 3.2 Estimation of causal effects of 41 inflammatory proteins with the risk of ED

**FIGURE 2:**
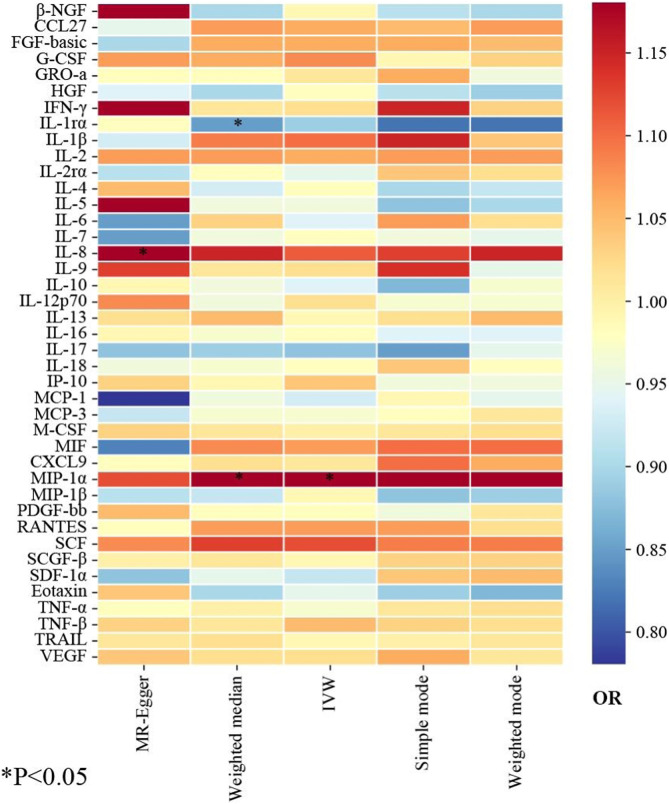
The heatmaps of five Mendelian randomization analysis methods. Different color blocks represent different odds ratio values. OR, odds ratio.

**FIGURE 3:**
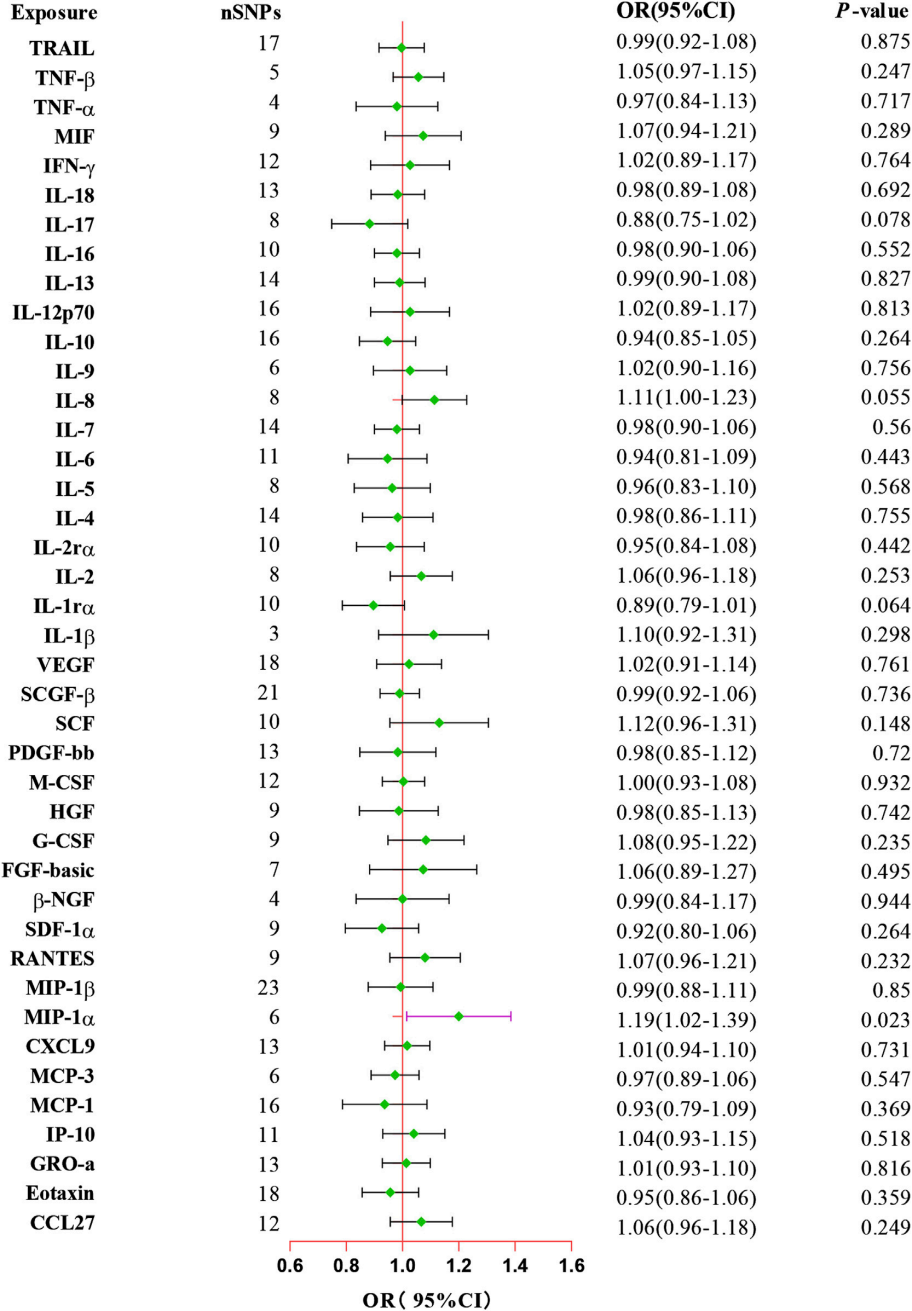
Forest plot of the Mendelian randomization analysis for the associations between systemic inflammatory proteins and erectile dysfunction. CI, confidence interval; OR, odds ratio. SNP, single nucleotide polymorphism.

**FIGURE 4:**
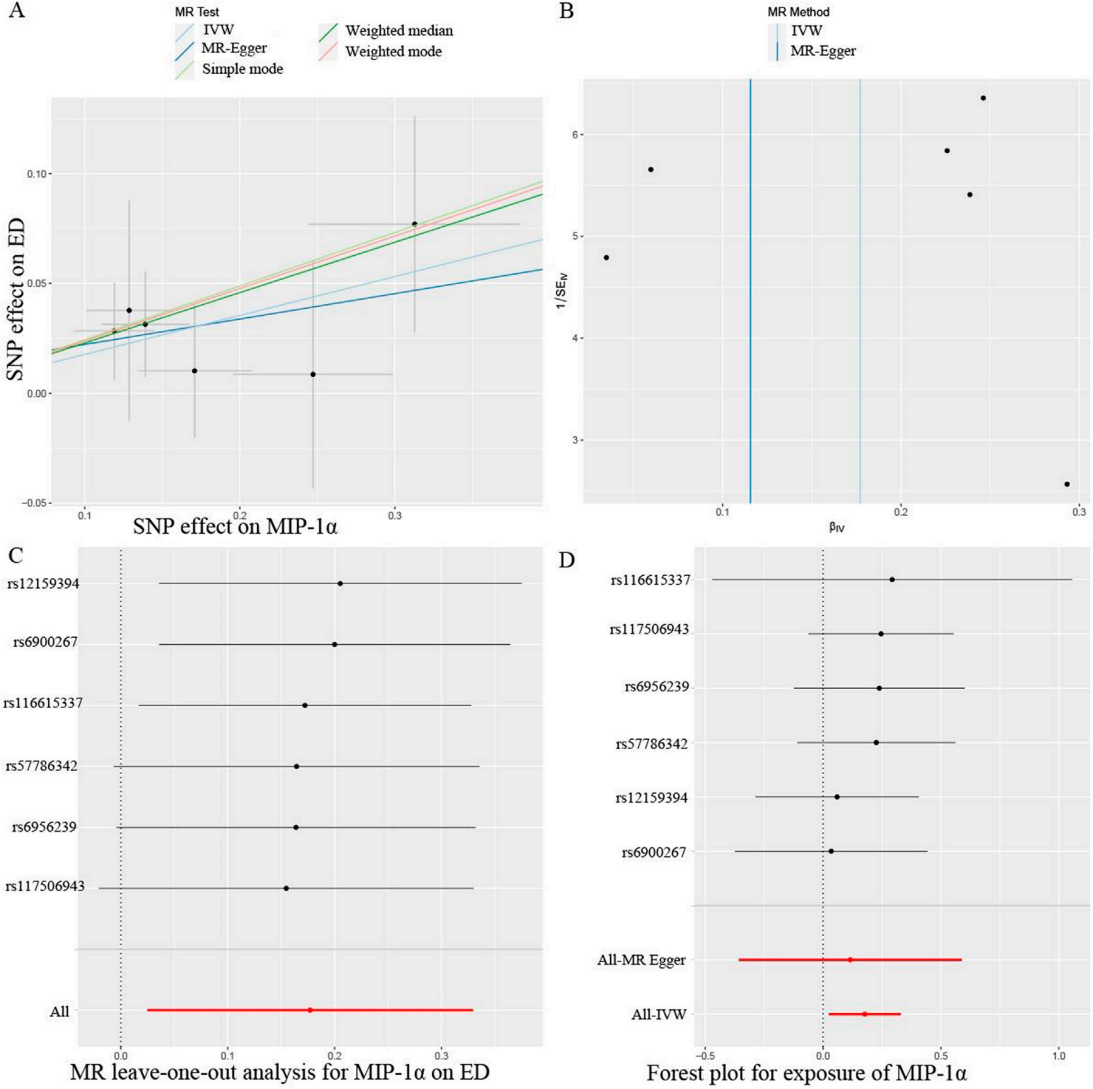
Mendelian randomization (MR) analyses for circulating levels of MIP-1α on erectile dysfunction (ED). **(A)** Scatter plot; **(B)** Funnel plot; **(C)** Leave-one-out analysis; **(D)** Forest plot. SNP, single nucleotide polymorphism.

The primary results from all MR analysis of the associations of 41 circulating inflammatory proteins with the risk of ED were displayed in Supplementary Table S3. To enhance the visual representation of IVs’ strength and facilitate a more intuitive understanding of the compiled data, we utilized heatmaps to present this aspect of the information (Figure 2). Additionally, the explicit estimates of the IVW approach were presented in Figure 3. After the Bonferroni correction, it was found by the IVW method that only genetically determined MIP-1α demonstrated suggestive an association with the risk of ED. The detailed information for the associated SNPs was summarized in Supplementary Table S4. Genetically predicted higher circulating levels of MIP-1α were suggestively associated with 19% higher odds for the risk of ED (OR:1.19, 95%CI:1.02 – 1.39, *p* = 0.023 by IVW method). It was consistent with the weighted median method (OR:1.26, 95%CI:1.04 – 1.52, *p* = 0.018). The results of the other methods revealed similar but not statistically significant trends (OR:1.12, 95%CI:0.70 –1.80, *p* = 0.657 by MR-Egger; OR:1.28, 95%CI:0.97 – 1.68, *p* = 0.140 by simple mode; OR:1.27, 95%CI:0.98 – 1.64, *p* = 0.126 by weighted mode). The scatter plot and funnel plot of Mendelian randomization analyses for MIP-1α on ED were presented in Figures 4A, B. MR-Egger regression analysis did not find underlying directional pleiotropy across the IVs (intercept *P*-value = 0.801). Furthermore, there was no evidence for heterogeneity in the association of MIP-1α as measured by Cochrane’s Q test (IVW: *p* = 0.927; MR-Egger: *p* = 0.86). The LOO sensitivity analysis and forest plot of Mendelian randomization analyses for MIP-1α on ED were shown in Figures 4C, D.

In addition, our MR analysis results showed suggestively negative effects of IL-1rα on the risk of ED (OR:0.85, 95%CI:0.72 – 1.00, *p* = 0.045 by weighted median), and IL-8 exhibited a suggestively positive association with the risk of ED (OR:1.26, 95%CI:1.05 – 1.50, *p* = 0.043 by MR-Egger method). However, the dominant IVW approach revealed that these two inflammatory proteins have no significant associations with the risk of ED (IL-1rα: OR:0.89, 95%CI:0.79 – 1.01, *p* = 0.064; IL-8: OR:1.11, 95%CI:1.00 – 1.23, *p* = 0.055). Additionally, the absence of evidence supported the lack of causal associations between other inflammatory proteins and the risk of ED (Supplementary Table S3). Moreover, MR-Egger and IVW Cochran’s Q statistics displayed that there was no significant heterogeneity of SNPs (all *P*-value > 0.05). For reverse MR analysis, we did not find statistically significant associations of ED with MIP-1α (OR:1.02, 95%CI:0.89 – 1.17, *p* = 0.738) utilizing the IVW method. The IVs we used and the detailed results of other methods are listed in Supplementary Table S5.

### 3.3 Molecular docking analysis

**FIGURE 5:**
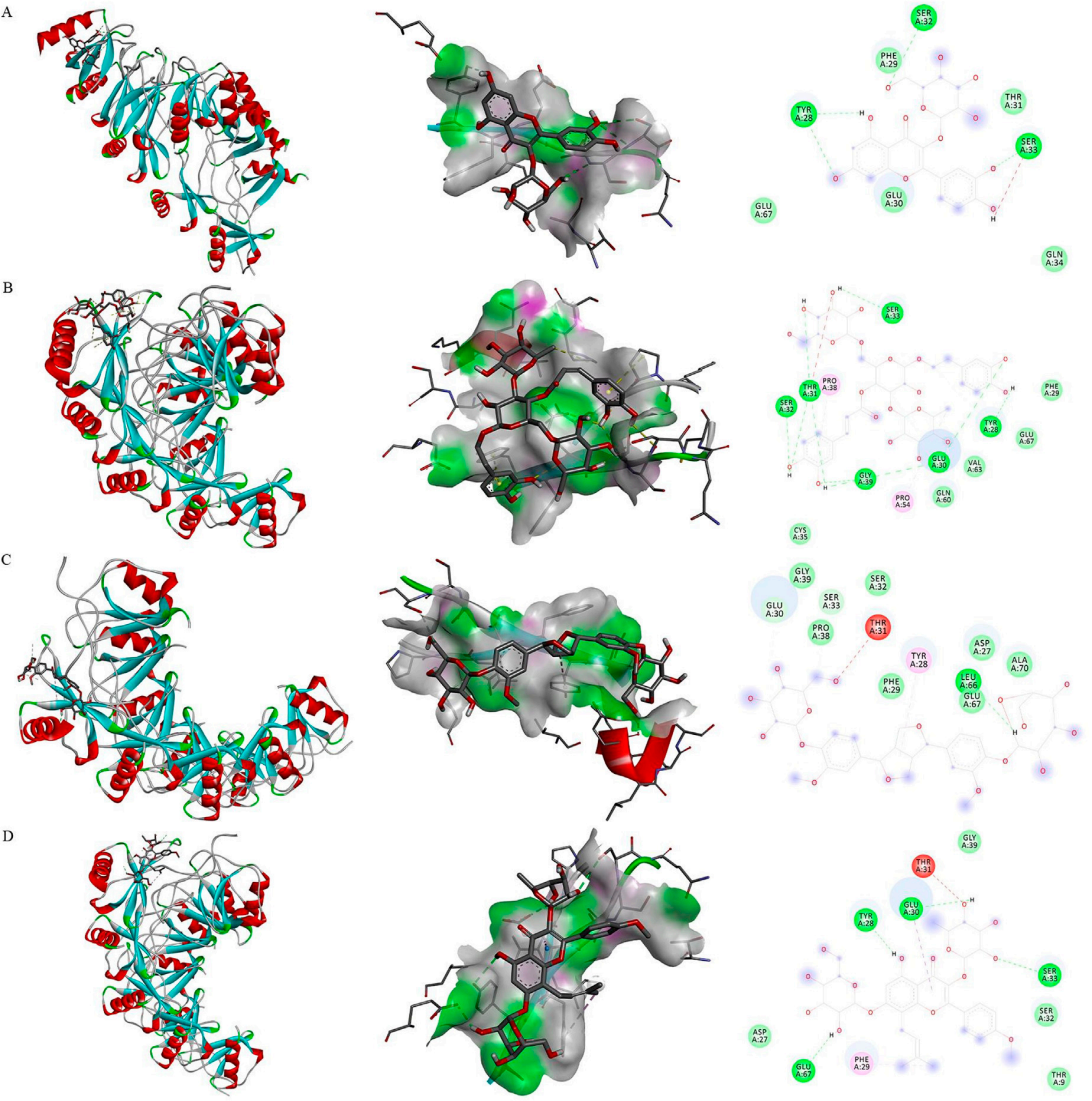
Molecular docking. Binding mode of proteins and ligands. **(A)** Binding mode of MIP-1α with hypericin; **(B)** Binding mode of MIP-1α with echinacea; **(C)** Binding mode of MIP-1α with pinoresinol diglucoside; **(D)** Binding mode of MIP-1α with icariin.

Based on the literature review, we selected the main active ingredients from the kidney-tonic, aphrodisiac and essence-enhancing herbs. Eventually, ten kinds of tonifying compounds, encompassing icariin, isopsoralen, nystose, pinoresinol diglucoside, ellagic acid, psoralen, protocatechuic acid, hypericin, catapol, and echinacea were identified. These compounds were selected as the key active ingredients to conduct molecular docking with MIP-1α. The key components and hub protein were validated by molecular docking utilizing the binding energy that reached or fell below −5.0 kcal/mol as the criterion (Feng et al., 2021). Eventually, the top four compounds were echinacea, pinoresinol diglucoside, hypericin, and icariin. The binding energy ranged from −5.9 to −5.0 kcal/mol, and the RMSD was less than 2, indicating that the significant active ingredients of anti-inflammatory or tonifying compounds were well with the hub targets. This suggests that the results of this study are reliable, as shown in Figures 5A–D and Supplementary Table S6.

### 3.4 PPI network construction of corresponding genes associated with MIP-1α

**FIGURE 6:**
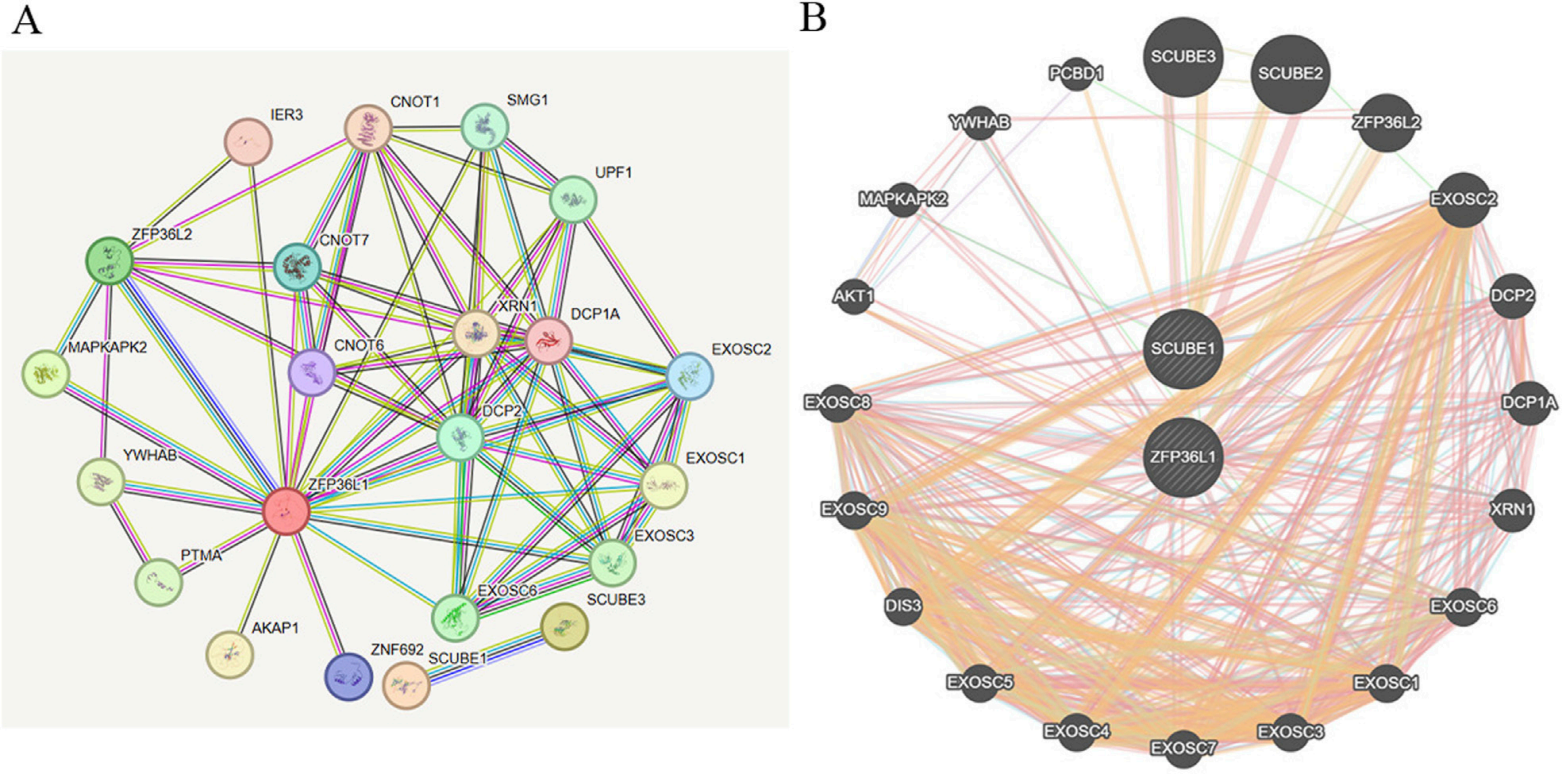
The construction of PPI network. **(A)** The hub genes visualization of PPI network by employing STRING database. **(B)** PPI network by utilizing GeneMANIA platform.

Altogether, we discovered that MIP-1α exhibited a suggestively causal association with ED. We employed the STRING online platform to establish a PPI network of overlapping hub genes, and then visualized the proteins interaction network by employing GeneMANIA software. Briefly, PPI findings revealed that the top 10 genes, encompassing SCUBE3, SCUBE2, ZFP36L2, EXOSC2, DCP2, DCP1A, XRN1, EXOSC6, EXOSC1, and EXOSC3 were primarily connected with the modulation and function of target gene associated with MIP-1α (Figures 6A, B).

## 4 Discussion

In this study, we firstly conducted multi-omics association study integrating GWAS and pQTL data to explore whether genetic evidence supported a causality of systemic inflammatory proteins with the risk of ED. Understanding the impact of inflammatory regulators in ED will provide us with further insights into the role inflammation plays in both the initiation and progression of ED. We observed a positive association between genetically predicted MIP-1α levels and the risk of ED, thereby providing molecular and epidemiological evidence supporting the potential role of targeted therapeutic drugs that regulate underlying inflammation in the treatment and prevention of ED. Additionally, we did not find any evidence indicating a causal association between ED and specific inflammatory protein through conducting reverse MR analysis. Meanwhile, for the candidate key protein MIP-1α, we utilized molecular docking technology to identify four anti-inflammatory or tonifying small molecular compounds, encompassing echinacea, pinoresinol diglucoside, hypericin, and icariin, which may be promising therapeutic targets for ED in the future.

To the best of our understanding, ED is an evolving health issue that causes sexual dysfunction in males (Zhu et al., 2023). The etiologies of ED encompass a range of factors, including endothelial dysfunction, atherosclerosis, and chronic inflammation (Li et al., 2018). Inflammation plays a significant pathophysiological role in the initiation and development of ED (Vlachopoulos et al., 2015). The presence of chronic low-grade inflammation plays a pivotal role in the pathogenesis of ED and is likely to be recognized as an intermediary stage for endothelial dysfunction (Kaya-Sezginer and Gur, 2020). Through augmented production of circulating proteins and upregulation of cellular adhesion molecules, the dysfunctional endothelium facilitates inflammation within the vascular wall, thereby establishing a conducive environment for the initiation and progression of atherosclerotic lesions in both penile vasculature and peripheral blood vessels (Vlachopoulos et al., 2007). Inflammation has been regarded as a shared factor in the pathogenesis of both ED and metabolic disorders. The inflammatory process of ED involves a complex modulation of cytokines, chemokines, and adhesion molecules (Kaya-Sezginer and Gur, 2020).

Macrophage inflammatory protein-1 alpha (MIP-1α), also referred to as CCL3, is a chemotactic cytokine possessing proinflammatory properties (Wu et al., 2019). MIP-1α has been reported to be vital for mediating inflammation responses (Noh et al., 2014; Zhang et al., 2016). MIP-1α could enhance inflammatory responses and augment the secretion of proinflammatory cytokines, such as IL-1β, TNF-α, and IL-6, which were synthesized by M1 macrophages (Chen et al., 2017). There are many studies on the correlations between MIP-1α and many relevant diseases of the genitourinary system. A previous animal experimental study conducted by Najjar et al. (2017) demonstrated that the accumulation of a specific subset of myeloid-derived suppressor cells in the renal cell carcinoma parenchyma was positively correlated with the intratumoral expression of MIP-1α. Another study conducted by Lopes and Callera (2012) suggested that the levels of IL-5, IL-4, IL-2, TNF-α, and MIP-1α were not significantly affected by irradiation in three-dimensional conformal radiotherapy of prostate cancer, while the levels of IL-6 seemed to reach their highest point after a duration of 15 days of radiotherapy. This evidence indicated that MIP-1α might be involved in the initiation and progression of urologic neoplasms. What’s more, the data from current research also revealed a positive association between serum levels of MIP-1α and IP-10, and this finding indicated that chemokines were involved in urinary tract inflammation of children (Gorczyca et al., 2014). However, the epidemiological evidence for the association between MIP-1α and ED was few, limited by small sample sizes, and restricted by using a case-control study design. In the present study, we observed that a higher genetically circulating level of MIP-1α was found to be significantly associated with an increased risk of ED. The evidence from experimental studies supported our findings. For instance, previous research conducted by P. Lu et al. reported that MIP-1α induced macrophages to infiltrate and produce VEGF by binding to CCR5 and eventually promoted angiogenesis (Lu et al., 2008). Although these studies demonstrated an association between circulating levels of MIP-1α and the risk of ED, the findings from these observational studies remained inconclusive and ambiguous. This situation was primarily attributed to the limited sample size and underlying confounding variables. Notably, our reverse MR analysis showed no associations between the risk of ED and serum MIP-1α levels. Thus, these results indicated that MIP-1α might be regarded as a potential therapeutic target for ED development. Nevertheless, given the indeterminacy of the findings, more studies are needed to validate the precise underlying biological mechanism of MIP-1α in the initiation and development of ED.

In our study, the associations between multiple inflammatory proteins and ED were assessed utilizing MR analysis. So far, several studies have explored the associations between partial inflammatory proteins and the risk of ED. Previously, J.K.Akintunde et al. reported that the erectile dysfunction caused by inflammation was associated with the alteration of NO-cGMP-dependent PKG signaling cascade through the upregulation of key cytokines and chemokine (MCP-1). Monocyte Chemoattractant Protein (MCP-1) could be considered for the management of inflammatory-mediated erectile dysfunction (IMED) (Akintunde et al., 2023). A previous study conducted by Unsworth et al. (2020) demonstrated that MCP-1 activation led to the expression of NF-kB, whereas inhibition of NF-kB has been shown to prevent inflammatory responses of TNF-α and MCP-1 in humans. These studies indicate that MCP-1 is a protective factor against ED, which goes in the same direction as our findings, although insignificant in statistics. Additionally, Vlachopoulos et al. (2006) conducted the research suggested that ED was related to elevated levels of circulating inflammatory proteins and endothelial-prothrombotic regulators. In his study, he found the presence of ED to be effectively ruled out by the detection of low levels of IL-6 and fibrinogen, as these simple biochemical substances demonstrated a satisfactory negative predictive value for ED. However, the findings of our study did not reveal any statistically significant impact of IL-6 on ED. This inconsistency in the results may be attributed to variations in sample size and diverse prevalence rates of ED across different populations. The role of TNF-α in inflammation-related ED is crucial, as it leads to an increase in arterial reactive oxygen species (ROS) and a reduction in NO levels (Carneiro et al., 2010). Hayward et al. reported that mice exhibiting human TNF-α overexpression manifested a reduction in erectile responses (Hayward et al., 2007). Moreover, the clinical research indicates an inverse association between TNF-α and sexual performance, with ED patients exhibiting higher serum levels of TNF-α independent of comorbid conditions (Karadag et al., 2007). These studies indicate that TNF-α exerts deleterious effects on erectile function. Intriguingly, for the association of TNF-α levels on the risk of ED, our findings suggest that there is no significant association between genetically predicted TNF-α and ED. These variations could be attributed to the diverse choice of IVs and GWAS summary data.

As previous studies described, the utilization of MR study has been introduced as a strategic approach to establish robust evidence for causal association between a biomarker and a disease (Mokry et al., 2015). Our study suggested that MIP-1α might be regarded as a potential therapeutic target or a biomarker for ED. The genetic factors that influence the biomarker will impact disease progression when the biomarker is a causal factor in disease progression (Khasawneh et al., 2022). Therefore, the identification of causal relationships between biomarkers and diseases by MR analysis renders them viable candidates for drug design (Khasawneh et al., 2022). However, examples are scarce in the literature regarding prospective studies that have utilized MR for drug development. In our study, we first employed molecular docking techniques to validate the interactions between the hub proteins MIP-1α and anti-inflammatory or tonifying compounds, as well as to screen potential therapeutic targets for ED. Through molecular docking, we found that the important active ingredients of echinacea, pinoresinol diglucoside, hypericin, and icariin combined well with the hub targets MIP-1α.

Inevitably, our study was subject to several limitations. First, the selection of IVs was conducted using a relaxed significance threshold of *P* < 5e-06, and this might lead to biased findings and false-positive variants. Nonetheless, the F-statistics of IVs all exceeded 10, indicating minimal presence of weak instrument bias. Similarly, several previous studies have also utilized the identical threshold (*P* < 5e-06) when assessing the associations between 41 inflammatory regulators and hypothyroidism (Lai et al., 2023). Second, the participants of GWAS included in the present study were limited to European descent, and it remains highly questionable whether the same results can be extrapolated to other races. Third, although we cannot eliminate the likelihood of pleiotropy, we have taken measures to exclude SNPs associated with potential confounding factors and performed multiple sensitivity analyses with different assumptions, such as MR-Egger regression. Fourth, after applying the Bonferroni correction, no cytokines demonstrated statistically significant correlations with ED, and only one of them (MIP-1α) showed suggestive associations. Fifth, when we explored the associations of IL-1rα and IL-8 with ED, the MR-Egger or weight-median estimates were significant, but the higher statistical power IVW approach was not statistically significant, this inconsistency could be attributed to the different choice of IVs and GWAS summary data. Lastly, the activities of inflammatory proteins are intricate, particularly those that exhibit high pleiotropy and possess the ability to exert their effects on diverse cell types. Additionally, circulating inflammatory proteins rarely demonstrate their effects alone but rather perform in regulatory networks (Ollier, 2004). Thus, these underlying associations need to be validated in larger cohorts and the potential involvement of cytokines in the regulation of ED development should be further investigated in future studies.

## 5 Conclusion

To sum up, our study provided support for the underlying causal association between circulating inflammatory protein MIP-1α and ED. Additionally, we utilized the molecular docking technology to identify four tonifying small molecular compounds, encompassing echinacea, pinoresinol diglucoside, hypericin, and icariin. Nevertheless, in the future, further investigations are warranted to validate these findings, elucidate potential biological mechanisms, and assess their utility as biomarkers and potential therapeutic targets for ED development.

## Data Availability

The original contributions presented in the study are included in the article/Supplementary Material, further inquiries can be directed to the corresponding author.
