# Supplementary material for: Multi-omics association study integrating GWAS and pQTL data revealed MIP-1α as a potential drug target for erectile dysfunction
Source: Front Pharmacol. 2024 Nov 1;15:1495970. doi: 10.3389/fphar.2024.1495970 (PMC11565697; doi:10.3389/fphar.2024.1495970)
Supplement: Supplementary file 1 [file DataSheet1.PDF]

Supplementary Materials:

Table S1. Brief characteristics description of systemic inflammatory proteins and erectile dysfunction GWAS cohorts involved in this study.

Table S2. Details of the number of genetic instruments and *F*-statistic for each circulating inflammatory protein.

Table S3. Effect estimates of the associations between each inflammatory protein and erectile dysfunction in MR analysis.

Table S4. Characteristics of instrumental variables used for circulating level of MIP-1α in this study.

Table S5. Effect estimates of the association of erectile dysfunction with circulating level of MIP-1α.

Table S6. Free binding energy and RMSD of the key active components of anti-inflammatory or tonifying compounds and key targets (MIP-1α).

Table S1. Brief characteristics description of systemic inflammatory proteins and erectile dysfunction GWAS cohorts involved in this study.

Exposure or outcome	Source	Sample size	Ancestry	Access Link	PMID
41 circulating inflammatory proteins	Finn Gen	8,293 participants	European	http://computationalmedicine.fi/data#Cytokine_GWAS	27989323
Erectile dysfunction	UK Biobank	6,175 cases and 217,630 controls	European	http://gwas.mrcieu.ac.uk/datasets/ebi-a-GCST006956/	30583798

Table S2. Details of the number of genetic instruments and *F*-statistic for each circulating inflammatory protein. ($P < 5 \times 10^{-6}$)

Inflammatory Proteins	Abbreviations	No. of SNPs	*F*-statistic (range)	Number
Cutaneous T-cell attracting (CCL27)	CTACK	12	22.92(20.84-31.44)	GCST004420
Beta nerve growth factor	NGF	4	24.59(20.83-35.43)	GCST004421
Vascular endothelial growth factor	VEGF	18	28.48 (20.88-71.72)	GCST004422
Macrophage migration inhibitory factor (glycosylation-inhibiting factor)	MIF	9	23.29(21.07-27.07)	GCST004423
TNF-related apoptosis inducing ligand	TRAIL	17	50.81 (20.83-345.00)	GCST004424
Tumor necrosis factor-beta	TNF-β	5	25.31 (20.98-38.27)	GCST004425
Tumor necrosis factor-alpha	TNF-α	4	22.78 (21.74-23.54)	GCST004426
Stromal cell-derived factor-1 alpha (CXCL12)	SDF-1α	9	22.51 (20.84-27.84)	GCST004427
Stem cell growth factor beta	SCGF-β	21	24.15 (20.83-42.10)	GCST004428
Stem cell factor	SCF	10	22.99 (21.13-25.82)	GCST004429
Interleukin-16	IL-16	10	28.99(21.21-53.45)	GCST004430
Regulated on activation, normal T Cell expressed and secreted (CCL5)	RANTES	9	23.01(20.84-27.21)	GCST004431
Platelet derived growth factor BB	PDGF-bb	13	23.74(21.15-31.12)	GCST004432

Macrophage inflammatory protein-1β (CCL4)	MIP-1β	23	28.34(20.95-87.99)	GCST004433
Macrophage inflammatory protein-1α (CCL3)	MIP-1α	6	22.04(20.94-24.11)	GCST004434
Monokine induced by interferon-gamma (CXCL9)	MIG	13	23.71(21.04-30.58)	GCST004435
Macrophage colony-stimulating factor	MCSF	12	21.96(20.92-25.52)	GCST004436
Monocyte specific chemokine 3 (CCL7)	MCP-3	6	22.21(20.77-26.39)	GCST004437
Monocyte chemotactic protein-1 (CCL2)	MCP-1	16	25.49(21.01-41.97)	GCST004438
Interleukin-12p70	IL-12p70	16	24.48(20.89-48.69)	GCST004439
Interferon gamma-induced protein 10 (CXCL10)	IP-10	11	22.32(20.87-24.72)	GCST004440
Interleukin-18	IL-18	13	23.59(21.29-27.31)	GCST004441
Interleukin-17	IL-17	8	23.85(20.90-32.13)	GCST004442
Interleukin-13	IL-13	14	22.63(20.87-25.78)	GCST004443
Interleukin-10	IL-10	16	30.76(20.98-132.61)	GCST004444
Interleukin-8 (CXCL8)	IL-8	8	21.95(20.89-23.85)	GCST004445
Interleukin-6	IL-6	11	22.77(20.77-30.71)	GCST004446
Interleukin-1 receptor antagonist	IL-1rα	10	21.94(20.77-24.25)	GCST004447
Interleukin-1-beta	IL-1β	3	22.15(21.79-22.74)	GCST004448
Hepatocyte growth factor	HGF	9	25.80(21.00-55.42)	GCST004449

Interleukin-9	IL-9	6	22.40(21.54-24.60)	GCST004450
Interleukin-7	IL-7	14	27.42(20.84-95.19)	GCST004451
Interleukin-5	IL-5	8	22.40(20.85-25.57)	GCST004452
Interleukin-4	IL-4	14	22.97(20.88-26.61)	GCST004453
Interleukin-2 receptor, alpha subunit	IL-2rα	10	28.06(20.93-77.94)	GCST004454
Interleukin-2	IL-2	8	22.80(20.94-27.62)	GCST004455
Interferon-gamma	IFN-γ	12	23.16(21.47-26.14)	GCST004456
Growth regulated oncogene-α (CXCL1)	GRO-α	13	22.97(20.83-29.89)	GCST004457
Granulocyte colony-stimulating factor	G-CSF	9	22.54(20.94-25.07)	GCST004458
Basic fibroblast growth factor	bFGF	7	22.04(20.79-25.40)	GCST004459
Eotaxin (CCL11)	Eotaxin	18	29.38(21.03-80.67)	GCST004460

Abbreviations: No., number; SNP, single nucleotide polymorphism.

Table S3. Effect estimates of the associations between each inflammatory protein and erectile dysfunction in MR analysis.

Methods	No. of SNPs	OR	95% CI	*P*-value	**P*-value
**NGF**					
MR-Egger	4	1.22	0.43-3.45	0.739	*0.727
Weighted median	4	0.90	0.73-1.11	0.317	
Inverse-variance weighted	4	0.99	0.84-1.17	0.944	
Simple mode	4	0.91	0.67-1.24	0.595	
Weighted mode	4	0.90	0.72-1.13	0.439	
**CTACK**					
MR-Egger	12	0.95	0.81-1.13	0.601	*0.153
Weighted median	12	1.07	0.94-1.21	0.299	
Inverse-variance weighted	12	1.06	0.96-1.18	0.249	
Simple mode	12	1.05	0.86-1.29	0.627	
Weighted mode	12	1.07	0.92-1.24	0.406	
**Eotaxin**					
MR-Egger	18	1.04	0.78-1.37	0.811	*0.536
Weighted median	18	0.90	0.90-1.04	0.167	
Inverse-variance weighted	18	0.95	0.86-1.06	0.359	
Simple mode	18	0.89	0.69-1.14	0.363	
Weighted mode	18	0.87	0.68-1.12	0.307	
**FGF-basic**					
MR-Egger	7	0.90	0.59-1.39	0.665	*0.448
Weighted median	7	1.06	0.83-1.34	0.651	
Inverse-variance weighted	7	1.06	0.89-1.27	0.495	
Simple mode	7	1.06	0.78-1.46	0.709	
Weighted mode	7	1.05	0.79-1.41	0.739	

**G-CSF**					
MR-Egger	9	1.07	0.90-1.28	0.469	*0.939
Weighted median	9	1.06	0.90-1.25	0.502	
Inverse-variance weighted	9	1.08	0.95-1.22	0.235	
Simple mode	9	0.99	0.77-1.29	0.967	
Weighted mode	9	1.03	0.84-1.26	0.793	
**GRO-a**					
MR-Egger	13	0.98	0.86-1.13	0.819	*0.644
Weighted median	13	0.98	0.87-1.10	0.768	
Inverse-variance weighted	13	1.01	0.93-1.10	0.816	
Simple mode	13	1.06	0.88-1.27	0.562	
Weighted mode	13	0.96	0.82-1.13	0.629	
**HGF**					
MR-Egger	9	0.94	0.69-1.27	0.678	*0.762
Weighted median	9	0.90	0.75-1.08	0.262	
Inverse-variance weighted	9	0.98	0.85-1.13	0.742	
Simple mode	9	0.91	0.71-1.17	0.482	
Weighted mode	9	0.89	0.73-1.08	0.269	
**IFN-γ**					
MR-Egger	12	1.29	0.99-1.67	0.089	*0.072
Weighted median	12	1.01	0.83-1.23	0.935	
Inverse-variance weighted	12	1.02	0.89-1.17	0.764	
Simple mode	12	1.15	0.82-1.60	0.438	
Weighted mode	12	1.03	0.78-1.37	0.834	
**IL-1rα**					
MR-Egger	10	0.98	0.69-1.38	0.903	*0.586

Weighted median	10	0.85	0.72-1.00	0.045	
Inverse-variance weighted	10	0.89	0.79-1.01	0.064	
Simple mode	10	0.82	0.66-1.01	0.092	
Weighted mode	10	0.82	0.65-1.03	0.126	
**IL-1β**					
MR-Egger	3	0.93	0.67-1.30	0.755	*0.457
Weighted median	3	1.09	0.88-1.36	0.421	
Inverse-variance weighted	3	1.10	0.92-1.31	0.298	
Simple mode	3	1.15	0.84-1.55	0.475	
Weighted mode	3	1.04	0.77-1.42	0.815	
**IL-2**					
MR-Egger	8	1.07	0.89-1.30	0.503	*0.910
Weighted median	8	1.07	0.93-1.24	0.335	
Inverse-variance weighted	8	1.06	0.96-1.18	0.253	
Simple mode	8	1.07	0.87-1.30	0.549	
Weighted mode	8	1.07	0.91-1.25	0.458	
**IL-2ra**					
MR-Egger	10	0.91	0.72-1.16	0.475	*0.690
Weighted median	10	0.98	0.86-1.12	0.805	
Inverse-variance weighted	10	0.95	0.84-1.08	0.442	
Simple mode	10	1.04	0.85-1.29	0.695	
Weighted mode	10	1.02	0.88-1.18	0.815	
**IL-4**					
MR-Egger	14	1.05	0.85-1.29	0.659	*0.435
Weighted median	14	0.93	0.79-1.09	0.362	
Inverse-variance weighted	14	0.98	0.86-1.11	0.755	

Simple mode	14	0.90	0.72-1.13	0.395	
Weighted mode	14	0.92	0.77-1.10	0.371	
**IL-5**					
MR-Egger	8	1.25	0.95-1.65	0.162	*0.083
Weighted median	8	0.96	0.81-1.14	0.622	
Inverse-variance weighted	8	0.96	0.83-1.10	0.567	
Simple mode	8	0.88	0.65-1.17	0.403	
Weighted mode	8	0.90	0.67-1.20	0.489	
**IL-6**					
MR-Egger	11	0.85	0.64-1.14	0.307	*0.439
Weighted median	11	1.03	0.85-1.26	0.749	
Inverse-variance weighted	11	0.94	0.81-1.09	0.443	
Simple mode	11	1.07	0.76-1.50	0.723	
Weighted mode	11	1.02	0.77-1.36	0.874	
**IL-7**					
MR-Egger	14	0.85	0.72-1.00	0.067	*0.075
Weighted median	14	0.96	0.86-1.06	0.385	
Inverse-variance weighted	14	0.98	0.90-1.06	0.560	
Simple mode	14	0.96	0.83-1.11	0.601	
Weighted mode	14	0.95	0.84-1.07	0.178	
**IL-8**					
MR-Egger	8	1.26	1.05-1.50	0.043	*0.131
Weighted median	8	1.15	1.00-1.31	0.053	
Inverse-variance weighted	8	1.11	1.00-1.23	0.055	
Simple mode	8	1.13	0.90-1.43	0.332	
Weighted mode	8	1.15	0.96-1.37	0.173	

**IL-9**					
MR-Egger	6	1.13	0.82-1.55	0.509	*0.548
Weighted median	6	1.01	0.85-1.21	0.912	
Inverse-variance weighted	6	1.02	0.90-1.16	0.756	
Simple mode	6	1.14	0.88-1.48	0.373	
Weighted mode	6	0.95	0.75-1.20	0.687	
**IL-10**					
MR-Egger	16	0.99	0.70-1.41	0.959	*0.768
Weighted median	16	0.96	0.83-1.11	0.586	
Inverse-variance weighted	16	0.94	0.85-1.05	0.264	
Simple mode	16	0.87	0.71-1.07	0.196	
Weighted mode	16	0.97	0.82-1.15	0.738	
**IL-12p70**					
MR-Egger	16	1.08	0.62-1.87	0.797	*0.836
Weighted median	16	0.96	0.80-1.15	0.656	
Inverse-variance weighted	16	1.02	0.89-1.17	0.813	
Simple mode	16	0.97	0.74-1.28	0.837	
Weighted mode	16	0.97	0.77-1.21	0.761	
**IL-13**					
MR-Egger	14	1.02	0.88-1.19	0.806	*0.638
Weighted median	14	1.05	0.93-1.19	0.425	
Inverse-variance weighted	14	0.99	0.90-1.08	0.827	
Simple mode	14	1.02	0.84-1.24	0.838	
Weighted mode	14	1.05	0.92-1.19	0.478	
**IL-16**					
MR-Egger	10	0.99	0.87-1.12	0.836	*0.834

Weighted median	10	0.97	0.87-1.07	0.507	
Inverse-variance weighted	10	0.98	0.90-1.06	0.552	
Simple mode	10	0.94	0.82-1.08	0.403	
Weighted mode	10	0.94	0.84-1.06	0.329	
**IL-17**					
MR-Egger	8	0.88	0.65-1.20	0.457	*0.941
Weighted median	8	0.89	0.73-1.08	0.236	
Inverse-variance weighted	8	0.88	0.75-1.02	0.087	
Simple mode	8	0.85	0.63-1.15	0.321	
Weighted mode	8	0.95	0.70-1.30	0.762	
**IL-18**					
MR-Egger	13	0.96	0.81-1.15	0.691	*0.824
Weighted median	13	0.97	0.87-1.09	0.661	
Inverse-variance weighted	13	0.98	0.89-1.08	0.692	
Simple mode	13	1.04	0.87-1.25	0.649	
Weighted mode	13	0.98	0.86-1.13	0.829	
**IP-10**					
MR-Egger	11	1.03	0.80-1.33	0.839	*0.953
Weighted median	11	0.99	0.86-1.13	0.831	
Inverse-variance weighted	11	1.04	0.93-1.15	0.518	
Simple mode	11	0.96	0.77-1.19	0.714	
Weighted mode	11	0.96	0.77-1.21	0.763	
**MCP-1**					
MR-Egger	16	0.78	0.52-1.19	0.269	*0.398
Weighted median	16	0.96	0.78-1.17	0.678	
Inverse-variance weighted	16	0.93	0.79-1.09	0.369	

Simple mode	16	0.99	0.69-1.42	0.959	
Weighted mode	16	0.95	0.71-1.28	0.746	
**MCP-3**					
MR-Egger	6	0.92	0.73-1.17	0.539	*0.651
Weighted median	6	0.97	0.87-1.08	0.587	
Inverse-variance weighted	6	0.97	0.89-1.06	0.547	
Simple mode	6	0.98	0.82-1.16	0.794	
Weighted mode	6	1.01	0.82-1.24	0.928	
**M-CSF**					
MR-Egger	12	1.03	0.88-1.20	0.730	*0.729
Weighted median	12	1.01	0.92-1.11	0.837	
Inverse-variance weighted	12	1.00	0.93-1.08	0.932	
Simple mode	12	1.01	0.88-1.16	0.875	
Weighted mode	12	1.02	0.91-1.16	0.722	
**MIF**					
MR-Egger	9	0.83	0.64-1.09	0.223	*0.081
Weighted median	9	1.08	0.92-1.28	0.345	
Inverse-variance weighted	9	1.07	0.94-1.21	0.289	
Simple mode	9	1.10	0.83-1.45	0.526	
Weighted mode	9	1.10	0.84-1.45	0.494	
**MIG**					
MR-Egger	13	0.98	0.84-1.14	0.801	*0.609
Weighted median	13	1.02	0.91-1.14	0.733	
Inverse-variance weighted	13	1.01	0.94-1.10	0.731	
Simple mode	13	1.10	0.91-1.32	0.352	
Weighted mode	13	1.06	0.91-1.24	0.477	

**MIP-1α**					
MR-Egger	6	1.12	0.70-1.80	0.657	*0.801
Weighted median	6	1.26	1.04-1.52	0.018	
Inverse-variance weighted	6	1.19	1.02-1.39	0.023	
Simple mode	6	1.28	0.97-1.68	0.140	
Weighted mode	6	1.27	0.98-1.64	0.126	
**MIP-1β**					
MR-Egger	23	0.91	0.67-1.25	0.584	*0.606
Weighted median	23	0.92	0.81-1.05	0.209	
Inverse-variance weighted	23	0.99	0.88-1.11	0.850	
Simple mode	23	0.88	0.70-1.12	0.318	
Weighted mode	23	0.89	0.74-1.07	0.235	
**PDGF-bb**					
MR-Egger	13	1.05	0.77-1.43	0.745	*0.595
Weighted median	13	0.98	0.82-1.16	0.792	
Inverse-variance weighted	13	0.98	0.85-1.12	0.720	
Simple mode	13	0.96	0.72-1.27	0.766	
Weighted mode	13	1.01	0.77-1.32	0.959	
**RANTES**					
MR-Egger	9	0.98	0.72-1.34	0.925	*0.572
Weighted median	9	1.07	0.92-1.24	0.404	
Inverse-variance weighted	9	1.07	0.96-1.21	0.232	
Simple mode	9	1.07	0.82-1.38	0.647	
Weighted mode	9	1.02	0.78-1.33	0.899	
**SCF**					
MR-Egger	10	1.08	0.77-1.53	0.667	*0.821

Weighted median	10	1.13	0.93-1.37	0.224	
Inverse-variance weighted	10	1.12	0.96-1.31	0.148	
Simple mode	10	1.09	0.79-1.51	0.612	
Weighted mode	10	1.09	0.77-1.54	0.646	
**SCGF-β**					
MR-Egger	21	1.00	0.87-1.16	0.969	*0.824
Weighted median	21	1.01	0.92-1.12	0.766	
Inverse-variance weighted	21	0.99	0.92-1.06	0.736	
Simple mode	21	1.03	0.87-1.22	0.768	
Weighted mode	21	1.03	0.89-1.20	0.676	
**SDF-1α**					
MR-Egger	9	0.88	0.70-1.11	0.319	*0.636
Weighted median	9	0.95	0.78-1.15	0.588	
Inverse-variance weighted	9	0.92	0.80-1.06	0.264	
Simple mode	9	1.04	0.76-1.44	0.809	
Weighted mode	9	1.05	0.80-1.38	0.734	
**TNF-α**					
MR-Egger	4	0.98	0.76-1.25	0.867	*0.976
Weighted median	4	1.00	0.84-1.19	0.999	
Inverse-variance weighted	4	0.97	0.84-1.13	0.717	
Simple mode	4	1.01	0.80-1.27	0.956	
Weighted mode	4	1.02	0.82-1.28	0.849	
**TNF-β**					
MR-Egger	5	1.03	0.87-1.22	0.748	*0.792
Weighted median	5	1.01	0.91-1.13	0.831	
Inverse-variance weighted	5	1.05	0.97-1.15	0.247	

Simple mode	5	1.03	0.89-1.18	0.739	
Weighted mode	5	1.02	0.90-1.15	0.790	
**TRAIL**					
MR-Egger	17	1.01	0.90-1.14	0.825	*0.639
Weighted median	17	1.02	0.92-1.13	0.712	
Inverse-variance weighted	17	0.99	0.92-1.08	0.875	
Simple mode	17	1.00	0.83-1.19	0.959	
Weighted mode	17	1.01	0.91-1.12	0.859	
**VEGF**					
MR-Egger	18	1.04	0.84-1.29	0.731	*0.824
Weighted median	18	1.02	0.88-1.17	0.818	
Inverse-variance weighted	18	1.02	0.91-1.14	0.761	
Simple mode	18	1.06	0.85-1.32	0.621	
Weighted mode	18	1.01	0.86-1.18	0.945	

Abbreviations: CI, confidence interval; OR, odds ratio; SNP, single nucleotide polymorphism.

**P*-value of the intercept from MR-Egger regression analysis.

Table S4. Characteristics of instrumental variables used for circulating level of MIP-1α in this study.

Inflammatory protein	SNP	Chr	Position	Closet gene	Effect allele	Beta	SE	*P*-value
MIP-1α	rs116615337	1	193533132	LOC124904475	A	0.1286	0.0278	3.66×10^{-06}
MIP-1α	rs117506943	11	80390225	NA	T	0.3128	0.0682	4.48×10^{-06}
MIP-1α	rs12159394	22	43638735	SCUBE1	A	-0.1708	0.0366	3.11×10^{-06}
MIP-1α	rs57786342	14	69260028	ZFP36L1	A	0.139	0.0283	8.91×10^{-07}
MIP-1α	rs6900267	6	380341	NA	A	-0.2472	0.0515	1.60×10^{-06}
MIP-1α	rs6956239	7	85345893	NA	T	0.119	0.026	4.58×10^{-06}

Abbreviations: Chr, chromosome; MIP-1α, macrophage inflammatory protein-1α (CCL3); SE, standard error; SNP, single nucleotide polymorphism.

Table S5. Effect estimates of the association of erectile dysfunction with circulating level of MIP-1α (reverse MR).

Methods	No. of SNPs	OR	95% CI	*P*-value（**P*-value）
**Erectile dysfunction (exposure)**				
MR-Egger	10	1.35	0.94-1.95	0.146（*0.152）
Weighted median	10	0.99	0.84-1.15	0.854
Inverse-variance weighted	10	1.02	0.89-1.17	0.738
Simple mode	10	1.00	0.79-1.28	0.977
Weighted mode	10	1.00	0.83-1.21	0.971

Abbreviations: CI, confidence interval; MIP-1α, Macrophage inflammatory protein-1α (CCL3); OR, odds ratio; SNP, single nucleotide polymorphism.; *P-value of the intercept from MR-Egger regression analysis.

Table S6. Free binding energy and RMSD of the key active components of anti-inflammatory or tonifying compounds and key target protein (MIP-1α).

**Component**	**Target**	**Free binding energy** (kcal/mol)	**RMSD**
Hypericin	MIP-1α	-5.6	0.925
Echinacea	MIP-1α	-5.9	1.344
Pinoresinol Diglucoside	MIP-1α	-5.8	0.625
Icariin	MIP-1α	-5.0	1.554

RMSD, Root Mean Square Deviation; MIP-1α, macrophage inflammatory protein-1α (CCL3).
